# Type I Kounis syndrome induced by COVID-19 vaccination in China: a case report

**DOI:** 10.1186/s12872-023-03289-6

**Published:** 2023-05-23

**Authors:** Yubing Deng, Zhujun Peng, Xiaoping Peng

**Affiliations:** 1 Department of Critical Care Medicine, Hunan Province Directly Affiliated TCM Hospital, Zhuzhou, China; 2 Department of Cardiovascular Medicine, Hunan Province Directly Affiliated TCM Hospital, Zhuzhou, China

**Keywords:** Kounis syndrome, COVID-19 vaccine, Allergic reactions, Acute coronary syndrome

## Abstract

**Background:**

Kounis syndrome is a rare clinical condition characterized by the occurrence of an acute coronary event induced by an acute allergic episode. The ongoing pandemic of coronavirus disease 2019 (COVID-19) has contributed to an increase in the incidence of allergic reactions to a certain extent, thereby increasing the incidence of Kounis syndrome. Timely diagnosis and effective management of this disease are important in clinical practice.

**Case presentation:**

We report a 43-year-old woman who developed generalized pruritus, breathlessness, paroxysmal precordial crushing pain, and dyspnea after receiving the third dose of the COVID-19 vaccine. After anti-allergic treatment and therapy for acute myocardial ischemia, her symptoms resolved with improvement in cardiac function and resolution of ST-segment changes. The prognosis was satisfactory, and the final diagnosis was type I Kounis syndrome.

**Conclusion:**

This patient with type I Kounis syndrome rapidly developed acute coronary syndrome (ACS) after an acute allergic reaction to the COVID-19 vaccine. ​Timely diagnosis of acute allergic reaction and ACS, and targeted treatment based on the relevant guidelines are the key to successful treatment of the syndrome.​

## Background

Kounis syndrome is a rare clinical condition that was first described by Kounis and Zavras in 1991. It is characterized by the occurrence of an acute coronary syndrome triggered by an allergic reaction to a drug or environmental allergen, resulting in the rupture of atheromatous plaque or vasospasm in the coronary arteries [1]. The pathogenesis of Kounis syndrome is not well elucidated. The main pathophysiological mechanism likely involves the occurrence of allergic reaction to external allergens, resulting in degranulation of mast cells and the release of large amounts of inflammatory mediators (e.g., histamine, proteases, prostaglandins, and leukotrienes) into the peripheral tissues. The consequent peripheral vasodilation and hypotension may affect coronary blood flow, leading to coronary spasm, plaque rupture, or intra-stent thrombosis, resulting in symptoms of myocardial ischemia [2]. Kounis syndrome may present as an acute allergic reaction (e.g., urticaria, angioneurotic edema, vomiting, abdominal pain, breathlessness, hypotension, shock), symptoms of acute myocardial ischemia (e.g., chest pain/discomfort), and palpitations, which may lead to heart failure, shock, or arrhythmia [3].

Despite the low incidence of the disease, the risk of cardiac involvement leading to heart failure, malignant arrhythmias, and sudden death is noteworthy. The ongoing pandemic of coronavirus disease 2019 (COVID-19) and the mass vaccination against this disease have likely contributed to an increased incidence of allergic reactions to some extent [4–6]. Therefore, exploration of the pathogenic mechanisms of Kounis syndrome and the development of countermeasures for clinical practice are key imperatives. In the present study, we report the first documented case of type I Kounis syndrome in China that developed after injection of the third booster dose of the COVID-19 vaccine.

## Case presentation

### The patient's condition before admission

A 43-year-old Chinese woman of Han ethnicity was transferred to our hospital because of chest tightness and pain in the precordial region at 16:00 Hrs on April 27, 2022, one hour after she received her third booster dose of the COVID-19 vaccine (Beijing KeXing ZhongWei, lot number: 202201004J) at a Community Health Service Center in Hunan Province, China. The pain was described as suffocating and crushing, and was associated with sweating and cold limbs, which appeared 40 min after vaccination (15:40 Hrs).

About 30 min before admission (30 min after vaccination, 15:30 Hrs), she developed generalized itch, accompanied by shortness of breath, nausea, and severe vomiting (watery vomitus consisting of coffee-colored gastric contents). Her vitals were: temperature 36.2℃; pulse rate 110 times/min; respiratory rate 26/min; blood pressure 89/50 mmHg. She was administered oxygen inhalation and anti-allergy treatment (intramuscular promethazine hydrochloride 25 mg; intravenous drip of dexamethasone 10 mg dissolved in 50 mL of normal saline). No vasopressor drugs were administered.​

The patient had no history of smoking or alcohol abuse, diabetes mellitus, or hypercholesterolemia.​ Her obstetric history included 4 pregnancies (1 live birth and 3 abortions). She had a history of developing skin rash after beer consumption, and a history of sudden-onset tinnitus after tetanus vaccination more than 10 years ago. ​Fifteen years ago, she was diagnosed with hepatitis B but was not found to have liver cirrhosis. Her previous medication history was unknown. On examination, her liver and spleen were not palpable, and laboratory tests showed normal liver function.​ There was no personal or family history of coronary artery disease.

### The patient's condition in the emergency department

**Fig. 1:**
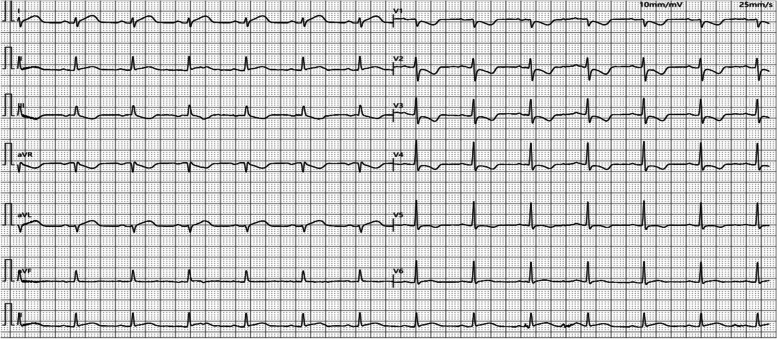
Emergency electrocardiogram of the patient performed on April 27, 2022 (sinus rhythm, both ST- elevation in leads I, II, and avL, and ST-segment depression and T-wave inversion in leads III and V1-V4)

**Fig. 2:**
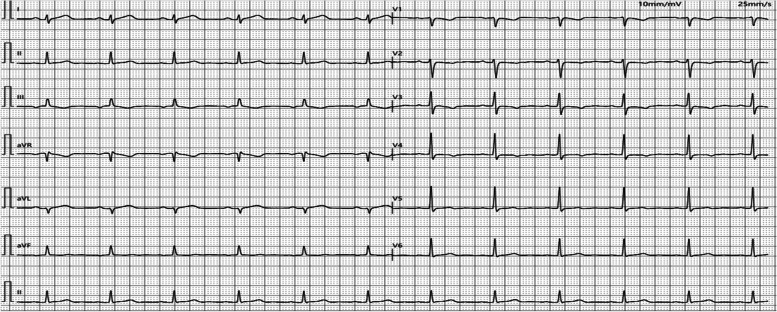
Repeated ECG performed on April 27, 2022 (sinus rhythm, ST-segment elevation in leads I, II, and avL has decreased compared to pre-treatment ECG. ​Compared to the previous ECG, the ST-T change indicates improved heart perfusion​)

Ten minites after admission (16:10 Hrs), the patient was found to be unconscious and was not responding to calls. She was pale, with cold skin and weak aortic pulsations. Her vitals were: temperature 36.0°C; pulse rate 115 times/min; respiratory rate 24 per min; and blood pressure 75/50 mmHg. An intramuscular injection of epinephrine (0.5 mg) was administered immediately along with intravenous infusion of Ringer's lactate solution. At 16:23 Hrs, her consciousness was restored, and her blood pressure increased to 85/52 mmHg. Electrocardiogram (ECG) showed sinus rhythm, ST-segment elevation in leads I, II, and avL, and ST-segment depression and T-wave inversion in leads III and V1-V4 (Fig. 1). At 16:44 Hrs, her serum cardiac troponin I (cTnI) level was 0.12 ng/mL (normal range, 0–0.028 ng/mL) and creatine kinase-MB (CK-MB) was 16.37 ng/mL (normal range, 0–25U/L). D-dimer, procalcitonin, interleukin-6, and C-reactive protein (CRP) levels were normal. COVID-19 nucleic acid test (throat swab) was negative. Bedside cardiac ultrasound revealed left atrial enlargement and left ventricular global systolic dysfunction with left ventricular ejection fraction (LVEF) of 29.1%. Echocardiography showed wall motion abnormalities namely a segmental hypokinesia in the lateral wall of left ventricle in the apical four-chamber view, consistent with ECG finding, suggesting the presence of anterior descending diagonal branch lesion. Chest auscultation revealed no wheezing; her heart sounds were weak and the possibility of heart failure was considered. The patient was administered dobutamine hydrochloride (15 µg/kg/min) via intravenous pump to maintain blood pressure along with moderate rehydration therapy. Repeated ECG performed at 17:19 Hrs displayed sinus rhythm and a decrease in the extent of ST-segment elevation in leads I, II, and avL compared with the previous ECG (Fig. 2). Compared to the previous ECG, the ST-T change indicated improved heart perfusion. Her blood pressure at 17:30 Hrs was 86/60 mmHg and a repeated intramuscular injection of 0.5 mg of epinephrine was administered. At 17:40 Hrs, her blood pressure had increased to 96/65 mmHg and she showed improved consciousness. She complained of intermittent chest tightness and breathlessness along with intermittent vomiting; she had a generalized punctate red-colored rash. Besides, her B-type natriuretic peptide (BNP) level was elevated (624.70 pg/mL; normal range, < 100 ng/mL), and her cTnI level was 0.16 ng/mL. Her myocardial enzyme spectrum was positive, and she was transferred to the intensive care unit (ICU) of our hospital.

### The patient's status in the ICU

Her physical parameters at admission to ICU were: body weight 60 kg; temperature 36.6°C, pulse rate 69 beats/min; respiratory rate 24 breaths/min; and blood pressure 90/58 mmHg maintained by intravenous administration of dopamine hydrochloride (15 µg/kg/min). On physical examination, she was drowsy and poorly cooperating. There were no signs of jaundice; scattered punctate red skin rashes were observed over the whole body, but there was no bleeding or petechiae. The pupils were bilaterally equal and round, with a dull light reflex. The mouth and lips were cyanotic. Chest auscultation showed bilateral coarse respiratory sounds with scattered wet rales without croup. The heart sounds were weak and rhythmic with no pathological cardiac murmur or pericardial friction sound.

**Fig. 3:**
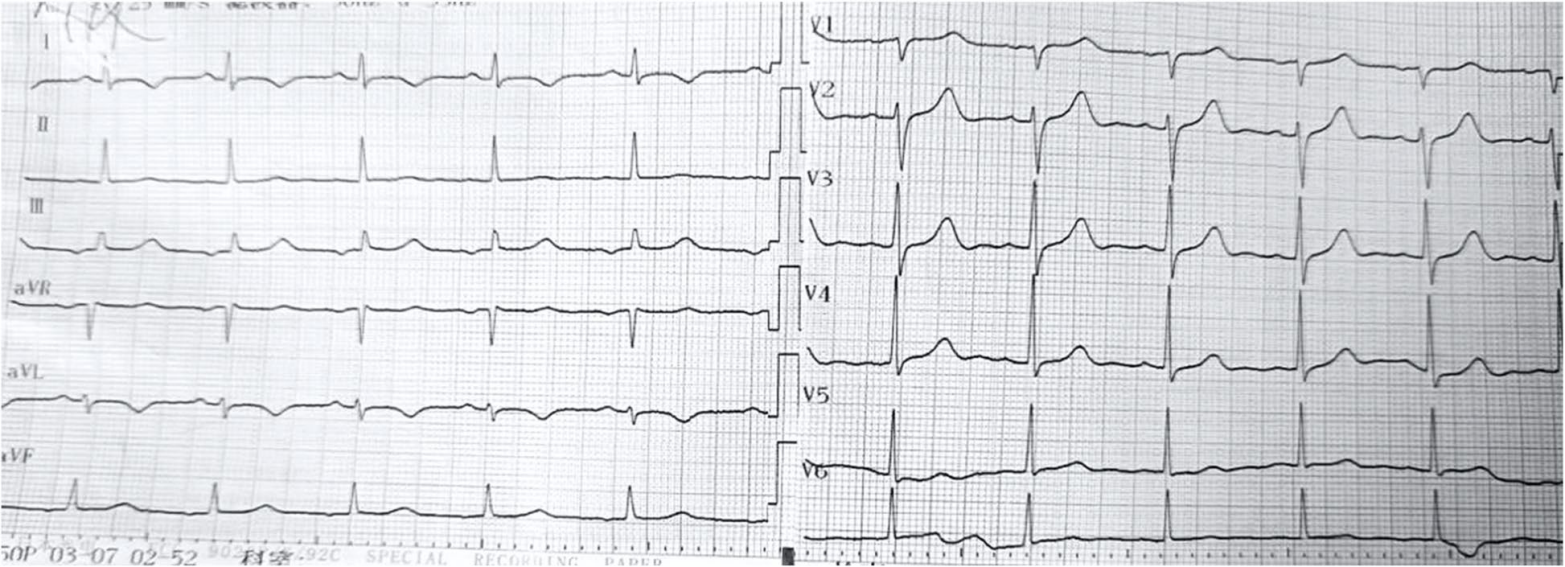
Repeated ECG performed on April 28, 2022 (sinus rhythm, ST-segment elevation in leads I, II, and avL fell back to baseline, T-wave inversion in leads I and avL, ST-segment depression in leads V1-V4 returned to baseline, and T-wave inversion became upright compared with pre-treatment ECG)

**Fig. 4:**
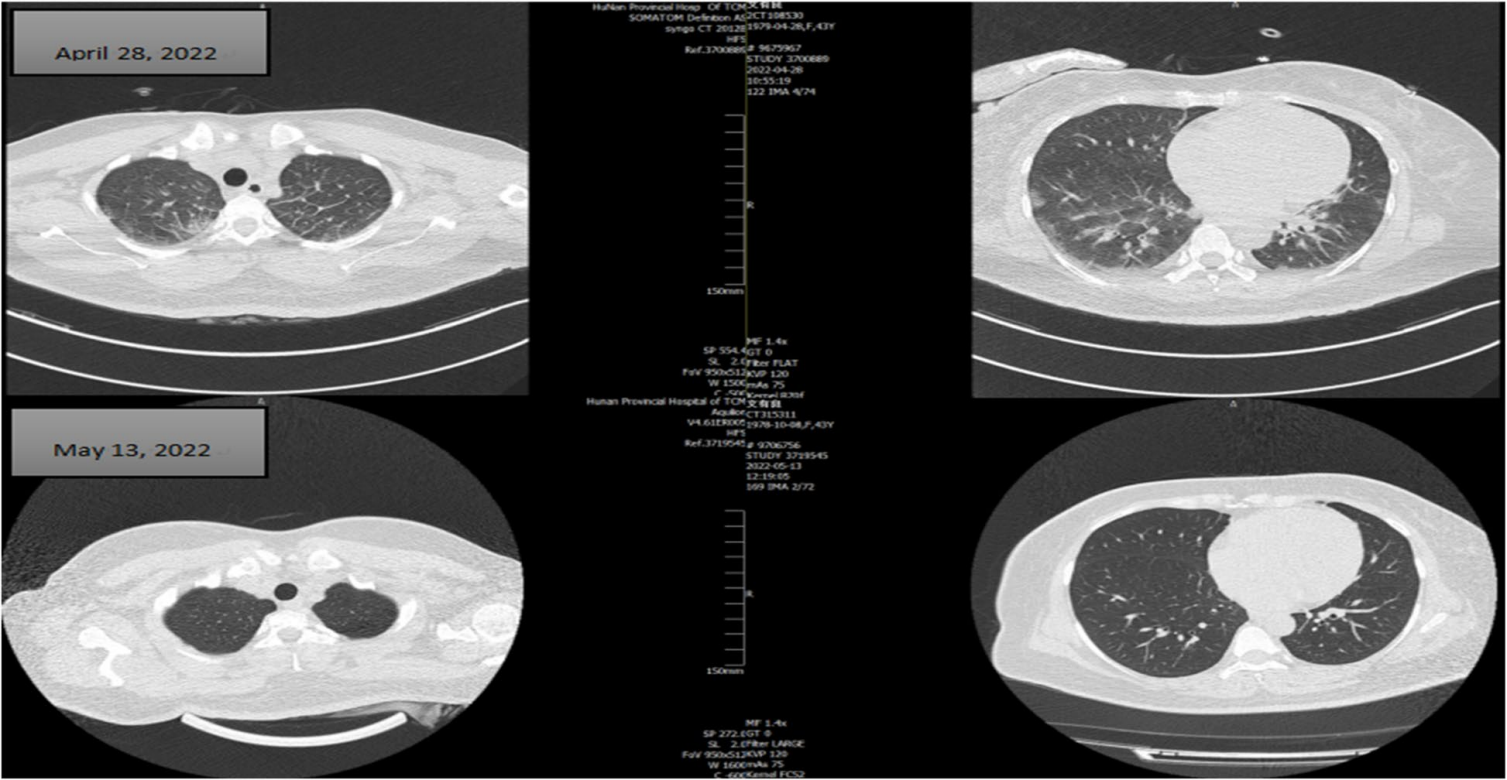
Lung CT on April 28, 2022, suggesting interstitial pulmonary edema; repeated lung CT on May 13, 2022 shows resolution of pulmonary edema

**Fig. 5:**
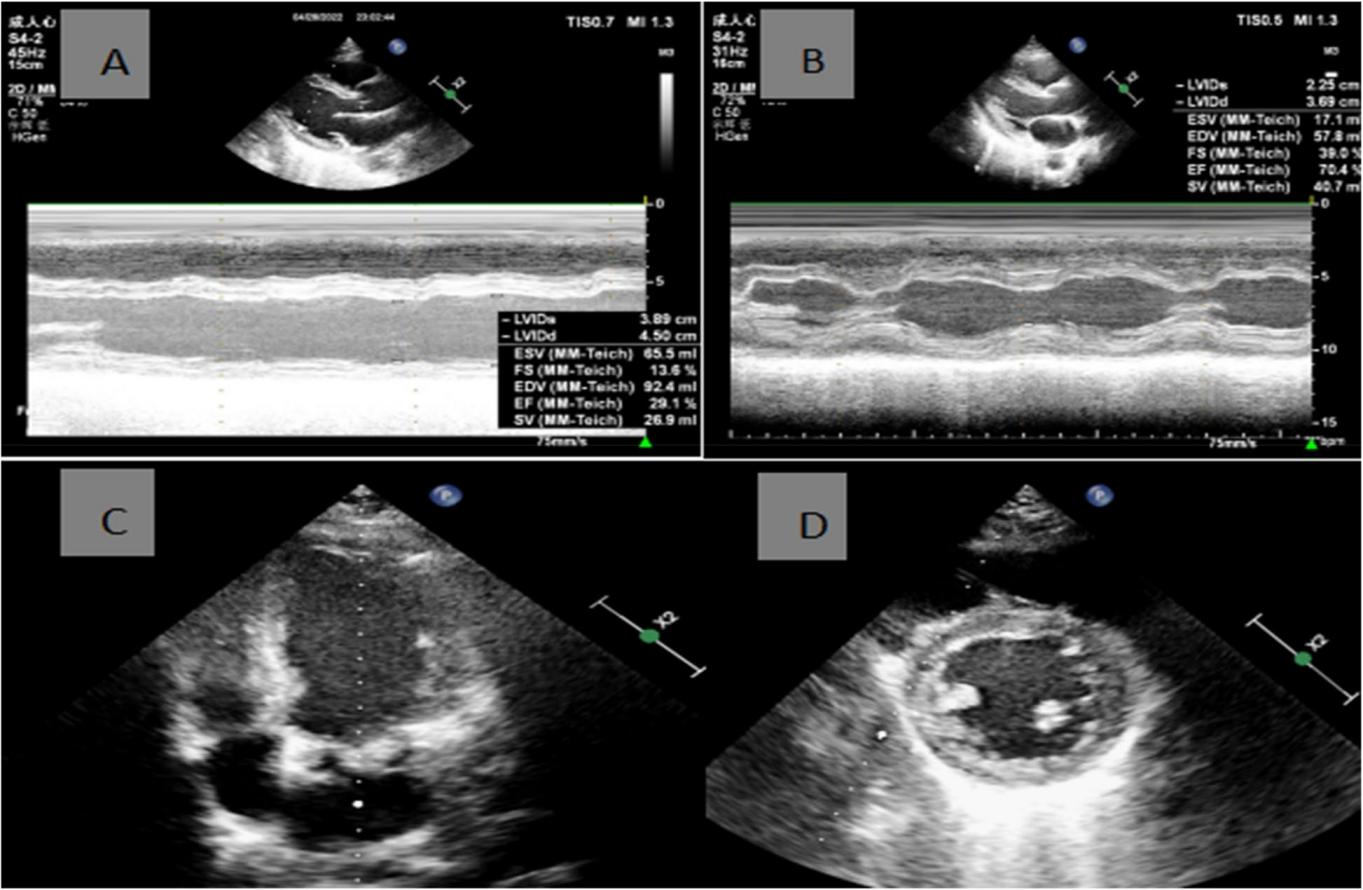
Cardiac ultrasound on April 28, 2022 (**A**): full left-sided heart (LA 35 mm/LV 55 mm), low left heart systolic function (LVEF, 29.1%; FS, 13.6%), mild mitral and tricuspid regurgitation. Repeated bedside cardiac ultrasound on May 16, 2022 (**B**) shows normal heart size and structure, as well as normal cardiac function (LVEF, 70%; FS, 39%). On April 28, 2022 (**C**/**D**), echocardiography showed wall motion abnormalities namely a segmental hypokinesia in the lateral wall of left ventricle in the apical four-chamber view, consistent with ECG finding, suggesting the presence of anterior descending diagonal branch lesion (considering that ECG STaVL↑>STI↑, the anterior descending diagonal branch lesion was more likely)

After admission to ICU, the relevant tests were completed, including ECG (Figs. 1, 2 and 3). Her serum primary cTnI was 0.12 ng/mL, the secondary was 0.16 ng/mL, and the tertiary was 2.02 ng/mL; mixed allergen panel indicated total immunoglobulin E (IgE)+, household dust mite ++, and beef + (Table 1). Lung computed tomography (CT) performed on April 28, 2022 showed signs of interstitial pulmonary edema (Fig. 4), which was significantly resolved on May 13, 2022 (Fig. 4). Bedside cardiac ultrasound performed on April 28, 2022 showed left atrium 35 mm, left ventricle 55 mm, low left heart systolic function [left ventricular ejection fraction (LVEF) 29.1%; fractional shortening (FS) 13.6%, mild mitral and tricuspid regurgitation] (Fig. 5). On May 16, 2022, she showed normal heart size and structure, as well as normal cardiac function (LVEF, 70%; FS, 39%). Of note, immediate percutaneous coronary intervention (PCI) was not performed because her family refused, owing to the allergic constitution of the patient and the high risk of an allergic reaction to the contrast agent.

**Table 1:** Allergen test results

Serial number	Item abbreviation	Item	Results
1	IgE	Total IgE	Positive
2	D1	House dust mites	Positive++
3	H1	House dust	Negative
4	E1	Cat dander	Negative
5	E5	Dog dander	Negative
6	W1	Short ragweed	Negative
7	T70	Mulberry	Negative
8	I6	Cockroach	Negative
9	Mx1	Penicillium notatum/ Cladosporium/ Aspergillus fumigatus/ Alternaria	Negative
10	Tx4	Oak tree/Elm tree/Phoenix tree/Willow tree/cottonwood/pollen	Negative
11	F202	Cashewhnut	Negative
12	F1	Egg white	Negative
13	F2	Milk	Negative
14	F3	Fish	Negative
15	F23	Crab	Negative
16	F24	Shrimp	Negative
17	F37	Shellfish	Negative
18	F27	Beef	Positive+
19	F88	Lamb	Negative
20	F91	Mango	Negative

1. Negative < 35 (IU/mL) No specific antibodies are detected.

2. + 0.35–0.70 (IU/mL) Very low titer antibodies are detected, usually asymptomatic but with some sensitivity.

3. ++ 0.70–3.50 (IU/mL) Low titer antibodies are detected, with certain sensitivity, and large amount of exposure usually results in clinical symptoms.

4. +++ 3.50–17.50 (IU/mL) Specific antibodies are detected, often accompanied by clinical symptoms.

5. ++++ 17.50–50.50 (IU/mL) High titer antibodies are detected, usually with clinical symptoms.

6. +++++ 50.50–100 (IU/mL) Very high antibody titers.

7. ++++++ >100 (IU/mL) Very high antibody titers.

### Treatment

**Fig. 6:**
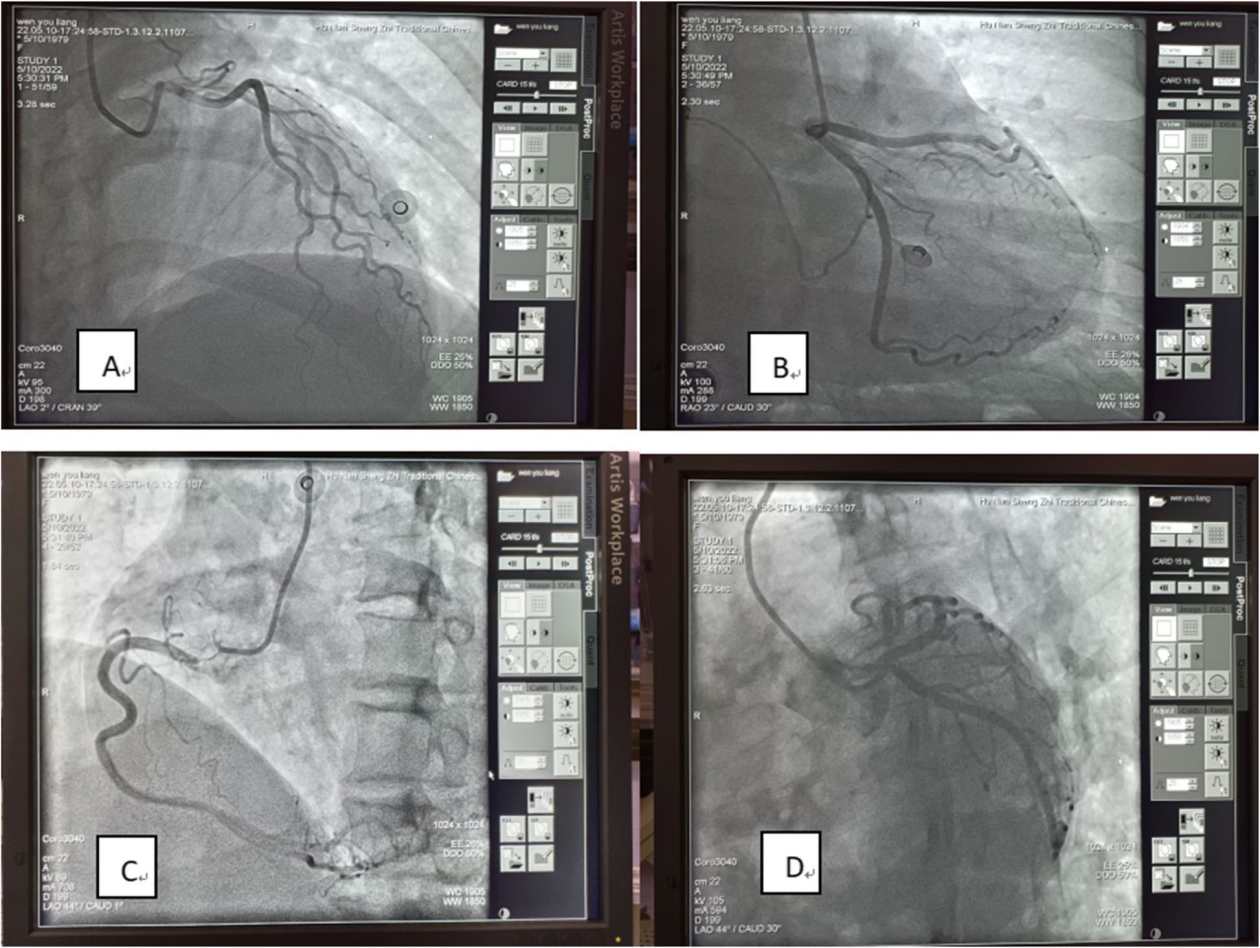
Elective coronary angiography findings of the patient on May 10, 2022 (Figure **A** (left coronary right cephalad)/Figure **B** (left coronary right pedicle)/Figure **C** (right coronary left anterior oblique)/Figure D (left coronary spider, showing no stenosis in the anterior descending, gyral branch, and right coronary arteries, with flow TIMI grade 3)

The treatment mainly included anti-allergic, anti-shock, and management of acute myocardial ischemia, including methylprednisolone, aspirin, clopidogrel, atorvastatin, low molecular weight heparin calcium subcutaneous injection, pantoprazole, nitroglycerin infusion, and maintenance of water-electrolyte acid-base balance. The patients’s symptoms resolved with the above-mentioned therapies, and the results of repeated bedside cardiac ultrasound, lung CT, myocardial enzyme spectrum, cTnI, and ECG findings were all normal. Elective coronary angiography performed on May 10, 2022 revealed normal coronary arteries (Fig. 6). There were no adverse reactions or accidents during hospitalization.

### Diagnosis

The patient was diagnosed with Type I Kounis syndrome. After vaccination, the patient developed an allergic reaction along with dyspnea and typical angina pectoris lasting up to 30 minutes. Myocardial enzyme profile and troponin levels were significantly increased, and the dynamic evolution of the electrocardiogram and cardiac ultrasound findings supported the diagnosis of acute myocardial infarction. Allergen profiling indicated an allergic constitution. The clinical picture was suggestive of a type II acute myocardial infarction due to allergy-induced spasm of the coronary arteries. The patients condition significantly improved after active treatment with anti-allergic, anti-coronary spasm, and anti-platelet therapy).​ The clinical picture was consistent with the diagnosis of type I Kounis syndrome.

### Follow up

​ The patient was discharged from the hospital on 13 May 2022 and has not experienced any discomfort during the follow-up period. On August 5, 2022, cardiac magnetic resonance imaging (CMR) showed no signs of myocarditis or myocardial edema, no spherical changes in the heart tip, and no significant abnormalities in systolic and diastolic function. ​A repeated ECG performed on 04 December 2022 showed no abnormalities.​

## Discussion and conclusion

### Diagnosis and differential diagnosis

The key points in the definition of type I Kounis syndrome are: 1) anaphylaxis leading to coronary artery spasm; 2) occurrence of myocardial infarction following the anaphylaxis, but with no evidence of myocardial ischemia prior to this episode [7]. The clinical presentation of the patient at the onset was consistent with anaphylaxis [8]. The patient developed shock symptoms 30 min after receiving the COVID-19 vaccine, and developed typical symptoms of myocardial ischemia 10 min later, indicating that anaphylactic shock preceded the onset of myocardial ischemia. The chest pain and electrocardiogram were consistent with the most recent diagnostic criteria for acute ST-segment elevation myocardial infarction [9, 10]. All these findings were consistent with the diagnosis of type I Kounis syndrome. Kounis syndrome is clinically classified into three types [7]. On the basis of anaphylactic damage, type I occurs in younger people without underlying native atherosclerotic artery disease. Type II occurs in those with preexisting atheromatous disease, and type III occurs in patients with coronary stents. Our case qualifies the criteria for type I.

### Distinguishing Kounis syndrome from Takotsubo cardiomyopathy

The differential diagnosis of Kounis syndrome includes Takotsubo cardiomyopathy because it can produce hyperkinesia of the ventricle base and hypokinesia of the apex and middle segments, which can also occur in Kounis syndrome as a result of inflammatory mediators. Takotsubo cardiomyopathy (also referred to as ‘broken heart syndrome’, ‘stress-induced cardiomyopathy’, and ‘apical ballooning syndrome’) is characterized by transient wall motion abnormalities of the left ventricle causing acute reversible heart failure that is not linked to obstructive coronary artery disease [11]. Stress cardiomyopathy often has a distinct cause. The patient had no signs of emotional distress at the time of vaccination, and there was no history of mood swings or adverse reactions after the previous two doses of the vaccine. Therefore, stress-induced cardiomyopathy was not likely. The main diagnostic tools used to distinguish TTS from ACS are echocardiography, coronary angiography, left ventricular angiography, and cardiac magnetic resonance imaging (CMR). The patient's echocardiography and CMR (August 5, 2022) showed no spherical changes in the apex, and no manifestations of myocardial edema. With an InterTAK Diagnostic Score [12] of 25, the probability of stress cardiomyopathy is only 0.3%. A previous in-depth study of the relevance of transthoracic echocardiography for the diagnosis of Takotsubo cardiomyopathy is of particular interest [11]. Our patient also underwent this examination and the imaging did not support a diagnosis of Takotsubo cardiomyopathy.

### Distinguishing Kounis syndrome from myocarditis after vaccination against COVID-19

Both conditions may be preceded by vaccination against COVID-19, and may present with chest pain, elevated troponin level, S-T segment elevation, heart failure, and cardiopulmonary shock. However, the two heart conditions have different mechanisms of onset. ​Kounis syndrome is myocardial ischemia or infarction secondary to coronary insufficiency due to anaphylactic shock immediately after vaccination, while the latter condition is caused by extensive myocardial damage due to an autoimmune reaction secondary to a second vaccination occurring more than one day later [13]. The time elapsed between the last vaccination and the onset of symptoms in our patients was 30 min, compared to 1–4 days in patients with COVID-19 mRNA vaccine-associated myocarditis [13]. Our patient had normal serum CRP levels, whereas 71–100% of patients with myocarditis after COVID-19 vaccination had elevated CRP levels [13].​ The patient had normal plasma levels of procalcitonin and interleukin-6, which also did not support a diagnosis of myocarditis.

### Other differential diagnosis

In terms of differential diagnosis, in addition to the cases of Takotsubo cardiomyopathy and myocarditis after vaccination against COVID-19 (hypersensitivity myocarditis), obstructive coronary artery disease (CAD), unstable angina, acute pericarditis, prinzmetal angina, eosinophilic coronary periarteritis, coronary allograft vasculopathy, and esophageal spasm should also be considered in the differential diagnosis [7]. However, the​ clinical presentation of our patient (acute hypersensitivity reaction immediately preceding the onset of ACS) along with the ECG, imaging, and laboratory findings ruled out the possibility of these conditions.​

There are some limitations in the diagnosis of this patient. Coronary angiography was not performed on time, considering that patients with allergic constitution may be allergic to contrast medium. Left ventriculography was not immediately performed. CT examination only clearly shows the condition of the lungs, but does not provide a direct diagnosis of the heart. CMR is useful for diagnosis and differential diagnosis [7, 11, 12], but was not performed in our patient on time.

### Treatment

Revascularization of the myocardium while managing allergic reactions is the mainstay of treatment for Kounis syndrome [7]. Treating the allergic reaction in the type I variant usually resolves the cardiac manifestations:

- Corticosteroids (hydrocortisone 1–2 mg/kg/d) suppress arterial hyperreactivity and relieve inflammation.
- H1 and H2 blockers (diphenhydramine 1–2 mg/kg and ranitidine 1 mg/kg) can also alleviate the allergic manifestations.
- Calcium channel blockers (CCB) and nitrates can help relieve the vasospasm induced by hypersensitivity and should be used if the blood pressure is satisfactory.
- Because epinephrine can worsen myocardial ischemia and induce coronary vasospasm and arrhythmias, it should be used with caution in KS. However, if the patient presents with anaphylaxis, the risks of untreated anaphylaxis outweigh the risk of possible worsening myocardial ischemia.​

The patient developed acute anaphylaxis and progressed to shock after being vaccinated prior to admission. She received intramuscular promethazine hydrochloride and intravenous dexamethasone. CCB was not used because of low blood pressure, which met the criteria for anti-allergy treatment.​ ACS occurred only 10 minutes after the onset of acute anaphylaxis and showed rapid progression. This was because the allergic reaction was so severe that the effects of the drugs given in the early stages could not keep up with the progress of the disease. ACS may not have occurred if rapid-acting epinephrine was administered immediately after the onset of acute anaphylaxis to correct shock.​

However, she regained consciousness 13 min after intramuscular administration of 0.5 mg epinephrine and her blood pressure increased to 85/52 mmHg, indicating that epinephrine acts rapidly in anaphylactic shock.​

After ICU admission, the patient's condition rapidly stabilized following systemic treatment as per ACS guidelines [9, 10]. In anaphylactic shock, there is dilation of the peripheral blood vessels, and nitroglycerin also dilates the peripheral blood vessels and reduces the amount of venous return. However, we gave the patient nitroglycerin. The patient suffered a myocardial infarction due to a severe allergy-induced coronary spasm, and the anaphylactic shock was corrected with anti-allergy therapy. Small doses of nitroglycerin may dilate coronary arteries and improve blood supply to the heart muscle when permitted by blood pressure monitoring and restoration of peripheral perfusion. In addition, moderate dilation of peripheral vessels reduces peripheral vascular resistance and cardiac after-load, and also improves cardiac function and peripheral perfusion.​ Another study also recommended nitroglycerin in this setting [7].

## Conclusion and experience from this case

Type I Kounis syndrome can develop rapidly after an acute allergic reaction caused by the COVID-19 vaccine, which may eventually lead to myocardial infarction. Although epinephrine may potentially aggravate coronary ischemia, timely use of epinephrine to treat anaphylactic shock may avert ACS.​ ​Timely diagnosis of acute allergic reactions and ACS and targeted treatment based on the latest evidence [7] and relevant guidelines [8–10] are the key to successful treatment of type I Kounis syndrome.​

## Data Availability

All relevant data supporting the conclusions of this article are included within the article.

## Abbreviations

ECG: Electrocardiogram
CT: Computed tomography
CMR: Cardiac magnetic resonance imaging

## References

[1] Yanagawa Y, Kondo A, Ishikawa K (2017). Kounis syndrome should be excluded when physicians treat patients with anaphylaxis[J]. Ann Allergy Asthma Immunol.

[2] Kounis NG, Cervellin G, Koniari I, Bonfanti L, Dousdampanis P, Charokopos N, Assimakopoulos SF, Kakkos SK, Ntouvas IG, Soufras GD, Tsolakis I. Anaphylactic cardiovascular collapse and Kounis syndrome: systemic vasodilation or coronary vasoconstriction? Ann Transl Med. 2018 ;6(17):332. 10.21037/atm.2018.09.05 PMC6174192 30306071

[3] Abdelghany M, Subedi R, Shah S, Kozman H. Kounis syndrome: a review article on epidemiology, diagnostic findings, management and complications of allergic acute coronary syndrome. Int J Cardiol. 2017;1:232:1–4. 10.1016/j.ijcard.2017.01.124 28153536

[4] Kounis NG, Koniari I, de Gregorio C, COVID-19 and Kounis Syndrome. Deciphering Their Relationship Balkan Med J. 2021;38(3):145–9. 10.5152/balkanmedj.2021.21097 PMC8880971 34142957

[5] Fialho I, Mateus C, Martins-Dos-Santos G, Pita J, Cabanelas N, Baptista SB, Roque D. Recurrent Kounis syndrome - a life-threatening event after COVID – 19 vaccine administration. J Cardiol Cases. 2022;25(6):400–3. 10.1016/j.jccase.2022.01.014 PMC8818380 35154518

[6] Sriwijitalai W, Wiwanitkit V. COVID-19 vaccine, myocardial infarction and Kounis syndrome. qjm. 2022;115(3):193. 10.1093/qjmed/hcac016 PMC9383371 35104343

[7] [Leen Alblaihed and Maite Anna Huis (2022). t Veld. Allergic Acute Coronary Syndrome—Kounis Syndrome. Emerg Med Clin North Am.

[8] Sampson HA, Mu ~ noz-Furlong A, Campbell RL, Adkinson NF Jr, Bock SA, Branum A et al. Second symposium on the definition and management of anaphylaxis: summary report–Second National Institute of Allergy and InfectiousDisease/Food Allergy and Anaphylaxis Network symposium. J Allergy Clin Immunol 2006;117:391-7. 10.1016/j.jaci.2005.12.1303 16461139

[9] Collet JP, Thiele H, Barbato E (2021). 2020 ESC Guidelines for the management of acute coronary syndromes in patients presenting without persistent ST-segment elevation. Eur Heart J.

[10] Ibanez B, James S, Agewall S (2018). 2017 ESC Guidelines for the management of acute myocardial infarction in patients presenting with ST-segment elevation: The Task Force for the management of acute myocardial infarction in patients presenting with ST-segment elevation of the European Society of Cardiology (ESC). Eur Heart J.

[11] Matta A, Delmas C, Campelo-Parada F, Lhermusier T, Bouisset F, Elbaz M, Nader V (2022). Stephanie Blanco, Jerôme Roncalli, Didier Carrié. Takotsubo cardiomyopathy. Rev Cardiovasc Med.

[12] Medina de Chazal H, Buono MGD, Keyser-Marcus L (2018). Stress Cardiomyopathy Diagnosis and Treatment. J Am Coll Cardiol.

[13] Biykem Bozkurt I, Kamat, Peter J. Hotez. Myocarditis With COVID-19 mRNA Vaccines.Circulation. 2021;144(6):471–484.: 10.1161/CIRCULATIONAHA.121.056135. 10.1161/CIRCULATIONAHA.121.056135 PMC8340726 34281357

